# Supplementary material for: Temperature-mediated dynamics: Unravelling the impact of temperature on cuticular hydrocarbon profiles, mating behaviour, and life history traits in three Drosophila species
Source: Heliyon. 2024 Aug 22;10(17):e36671. doi: 10.1016/j.heliyon.2024.e36671 (PMC11387341; doi:10.1016/j.heliyon.2024.e36671)
Supplement: Multimedia component 1 [file mmc1.docx]

|  |  | Df | Sum Sq | Mean Sq | F value | Pr(>F) |  |
| --- | --- | --- | --- | --- | --- | --- | --- |
| *D. ezoana* | | Temperatures | 3 | 103.46 | 34.49 | 48.159 | <0.0001 |
|  |  | Sex | 1 | 15.6 | 15.6 | 21.789 | <0.0001 |
|  |  | Temperatures * Sex | 3 | 13.4 | 4.47 | 6.237 | 0.000797 |
| *D. novamexicana* | | Temperatures | 3 | 164.56 | 54.85 | 43.653 | <0.0001 |
|  |  | Sex | 1 | 15.4 | 15.4 | 12.255 | 0.0008 |
|  |  | Temperatures * Sex | 3 | 14.17 | 4.72 | 3.758 | 0.0145 |
| *D. virilis* | | Temperatures | 3 | 602.3 | 200.76 | 30.235 | <0.0001 |
|  |  | Sex | 1 | 5.5 | 5.51 | 0.83 | 0.365 |
|  |  | Temperatures * Sex | 3 | 41.8 | 13.93 | 2.098 | 0.108 |

**Table 1:** Two-way ANOVA on the effects of temperature and sex on the adult body mass of *D. ezoana*, *D. novamexicana*, and *D. virilis*.

**Table 2:** Two-way ANOVA on the effects of temperature and sex on the adult dry body mass of *D. ezoana*, *D. novamexicana*, and *D. virilis*.

|  |  | Df | Sum Sq | Mean Sq | F value | Pr(>F) |
| --- | --- | --- | --- | --- | --- | --- |
| *D. ezoana* | Temperatures | 3 | 43.09 | 14.362 | 38.955 | <0.0001 |
|  | Sex | 1 | 6.85 | 6.852 | 18.584 | <0.0001 |
|  | Temperatures * Sex | 3 | 9.21 | 3.069 | 8.325 | <0.0001 |
| *D. novamexicana* | Temperatures | 1 | 6.85 | 6.852 | 18.584 | <0.0001 |
|  | Sex | 3 | 43.09 | 14.362 | 38.955 | <0.0001 |
|  | Temperatures * Sex | 3 | 9.21 | 3.069 | 8.325 | <0.0001 |
| *D. virilis* | Temperatures | 3 | 297.44 | 99.15 | 159.821 | <0.0001 |
|  | Sex | 1 | 4.94 | 4.94 | 7.955 | 0.00619 |
|  | Temperatures * Sex | 3 | 0.3 | 0.1 | 0.159 | 0.92326 |
