# Supplementary material for: Temperature-mediated dynamics: Unravelling the impact of temperature on cuticular hydrocarbon profiles, mating behaviour, and life history traits in three Drosophila species
Source: Heliyon. 2024 Aug 22;10(17):e36671. doi: 10.1016/j.heliyon.2024.e36671 (PMC11387341; doi:10.1016/j.heliyon.2024.e36671)
Supplement: Multimedia component 2 [file mmc2.docx]

**Supplementary Figures**


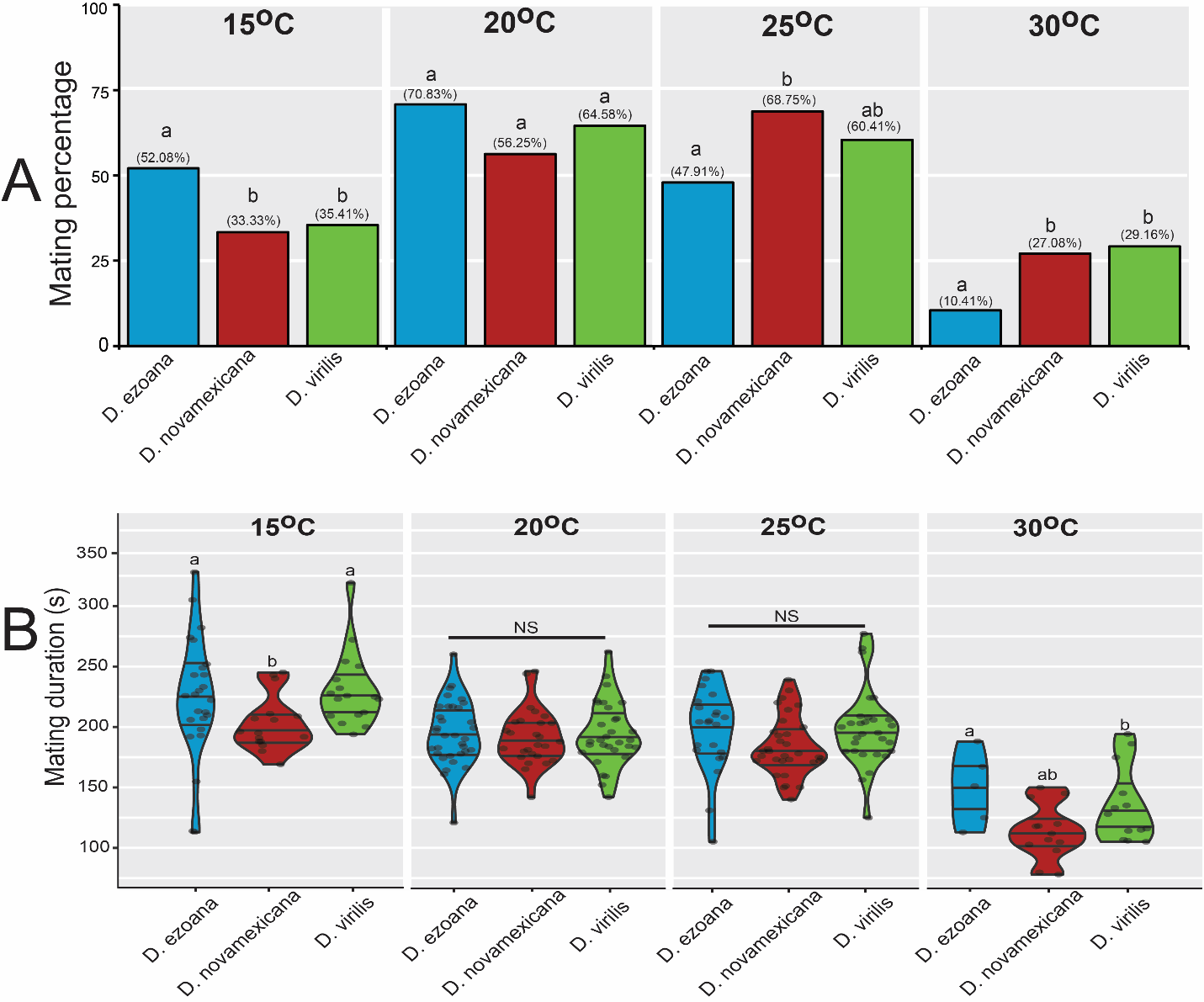


Supplementary figure 1. **The three *Drosophila* species exhibit distinct mating behavior**. (**A**) Bar graphs comparing the mating percentage of *D. ezoana*, *D. novamexicana* and *D. virilis* when developed at 15, 20, 25, and 30^o^C. (B) Violin plots comparing the mating time of *D. ezoana*, *D. novamexicana* and *D. virilis* when developed at 15, 20, 25, and 30^o^C. Dots on each violin plot indicate data points from each replicate. Distinct letters on each graph denote significant differences (Mating percentage: Chi-square test; Mating duration: ANOVA followed by the SNK posthoc tests).


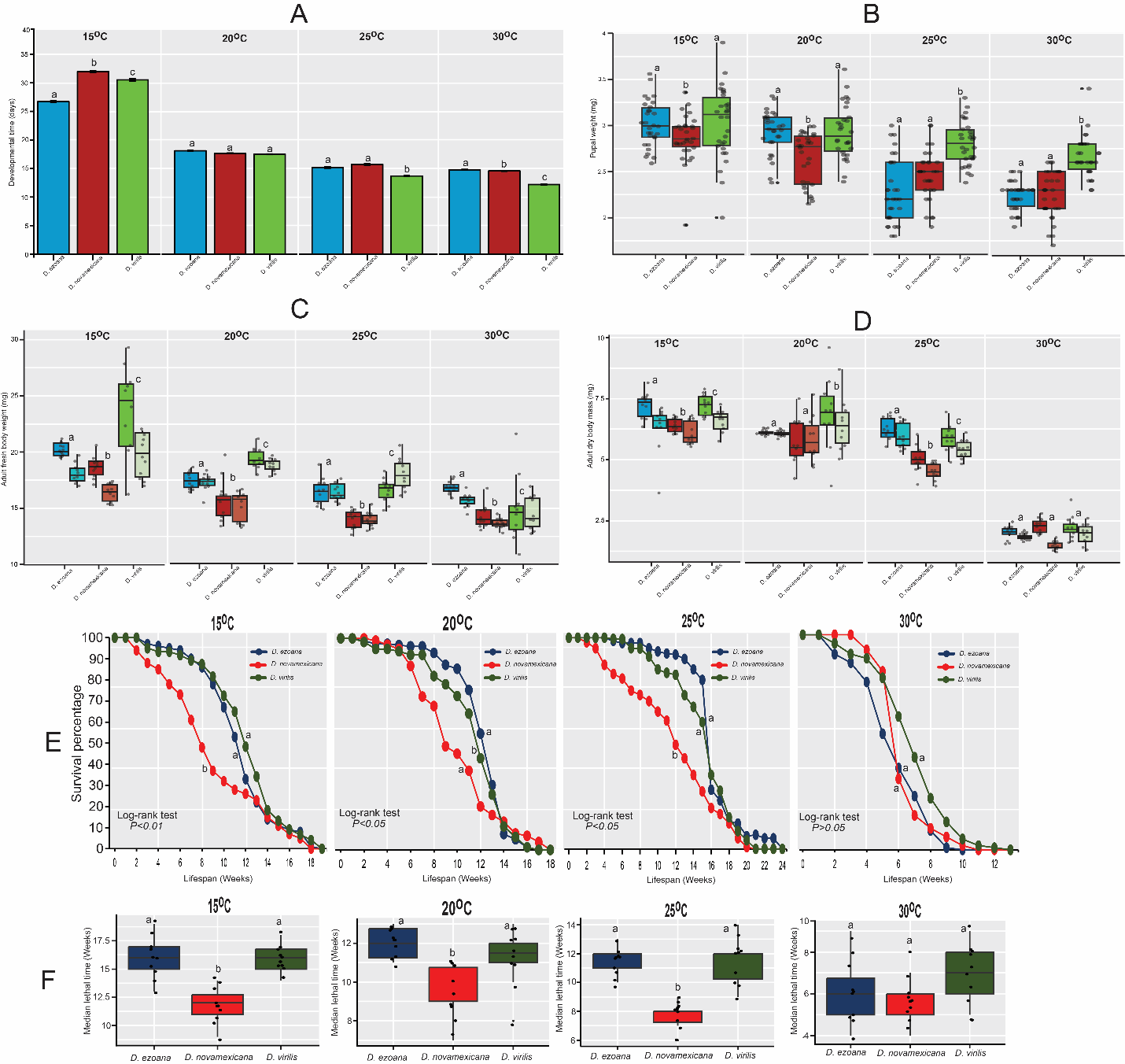


Supplementary figure 2. **The three *Drosophila* species exhibit different life history traits.** (**A**) Bar graphs comparing the mean developmental time of the offspring of *D. ezoana*, *D. novamexicana* and *D. virilis* when developed at 15°C, 20°C, 25°C, and 30°C. Error bars indicate standard error of the mean (SEM). Bars with different letters are significantly different from each other (ANOVA followed by the SNK posthoc tests; *P* < 0.05). (**B**) Boxplots comparing the pupae weight of *D. ezoana*, *D. novamexicana* and *D. virilis* when developed at 15°C, 20°C, 25°C, and 30°C. (**C**) Boxplots comparing the fresh adult (female and male) body weight of *D. ezoana*, *D. novamexicana* and *D. virilis* when developed at 15°C, 20°C, 25°C, and 30°C. (**D**) Boxplots comparing the dry adult (female and male) body weight of *D. ezoana* , *D. novamexicana* and *D. virilis* when developed at 15°C, 20°C, 25°C, and 30°C. (**E**) Kaplan-Meier curves comparing the variation of the longevity of adult offspring produced by *D. ezoana* , *D. novamexicana* (middle) and *D. virilis* when developed at 15°C, 20°C, 25°C, and 30°C. (**F**) Boxplots comparing the change of the median lethal time of adult offspring produced by *D. ezoana*, *D. novamexicana* and *D. virilis* when developed at 15°C, 20°C, 25°C, and 30°C. In each boxplot, the ends of boxplot whiskers represent the minimum and maximum values of all the data and dots show individual data points. Significant differences are among species are illustrated by different letters (ANOVA followed by the SNK posthoc tests; *P* < 0.05, n = 10).
